# Cold shock Y-box protein-1 proteolysis autoregulates its transcriptional activities

**DOI:** 10.1186/1478-811X-11-63

**Published:** 2013-08-27

**Authors:** Claudia RC van Roeyen, Florian G Scurt, Sabine Brandt, Vanessa A Kuhl, Sandra Martinkus, Sonja Djudjaj, Ute Raffetseder, Hans-Dieter Royer, Ioannis Stefanidis, Sandra E Dunn, Steven Dooley, Honglei Weng, Thomas Fischer, Jonathan A Lindquist, Peter R Mertens

**Affiliations:** 1 Department of Nephrology and Clinical Immunology, RWTH Aachen University, Aachen, Germany; 2 Department of Nephrology and Hypertension, Diabetes and Endocrinology, Otto-von-Guericke University Magdeburg, Leipziger Str 44, 39120 Magdeburg, Germany; 3 Breast Cancer Research, Center of Advanced European Studies and Research, Caesar, Bonn, Germany; 4 Department of Nephrology, University of Thessaly, Larissa, Greece; 5 Laboratory for Oncogenomic Research, Departments of Pediatrics and Experimental Medicine, Child and Family Research Institute, University of British Columbia, Vancouver, British Columbia, Canada; 6 Molecular Hepatology, Department of Medicine II, University of Heidelberg, Mannheim, Germany; 7 Department of Hematology and Oncology, Otto-von-Guericke University Magdeburg, Magdeburg, Germany

**Keywords:** Cold shock protein, DbpB, YBX1, Nuclear localization signal, Post-translational modification, RNA/DNA binding protein

## Abstract

**Background:**

The Y-box protein-1 (YB-1) fulfills pleiotropic functions relating to gene transcription, mRNA processing, and translation. It remains elusive how YB-1 shuttling into the nuclear and cytoplasmic compartments is regulated and whether limited proteolysis by the 20S proteasome releases fragments with distinct function(s) and subcellular distribution(s).

**Results:**

To address these questions, mapping of domains responsible for subcellular targeting was performed. Three nuclear localization signals (NLS) were identified. NLS-1 (aa 149-156) and NLS-2 (aa 185-194) correspond to residues with unknown function(s), whereas NLS-3 (aa 276-292) matches with a designated multimerization domain. Nuclear export signal(s) were not identified. Endoproteolytic processing by the 20S proteasome before glycine 220 releases a carboxy-terminal fragment (CTF), which localized to the nucleus, indicating that NLS-3 is operative. Genotoxic stress induced proteolytic cleavage and nuclear translocation of the CTF. Co-expression of the CTF and full-length YB-1 resulted in an abrogated transcriptional activation of the MMP-2 promoter, indicating an autoregulatory inhibitory loop, whereas it fulfilled similar trans-repressive effects on the collagen type I promoter.

**Conclusion:**

Compartmentalization of YB-1 protein derivatives is controlled by distinct NLS, one of which targets a proteolytic cleavage product to the nucleus. We propose a model for an autoregulatory negative feedback loop that halts unlimited transcriptional activation.

## Background

Cold shock proteins (CSP) are amongst the most conserved proteins in evolution, sharing a cold shock domain (CSD) from pro- to eukaryotes [1]. Numerous functions have been unravelled for members of this protein family. In bacteria CSPs are co-ordinately up-regulated following a decrease in temperature to rescue bacterial growth [2]. In eukaryotic cells CSPs are involved in the transcriptional regulation of genes related to cell proliferation (e.g. DNA polymerase-α [3], cyclins A and B1 [4], FAS receptor [5]). Further target genes coordinate matrix synthesis and degradation [6], inflammatory responses (e.g. IL-2 [7], GM-CSF [8]), and antigen presentation (major human leukocyte antigen [9], ABC transporters [10]).

Y-box protein (YB)-1 is the prototypic member of the cold shock protein family in humans. YB-1 acts in a cell-context specific manner on gene transcription, e.g. of matrix-metalloproteinase (MMP)-2 [11] and granulocyte macrophage-colony stimulating factor (GM-CSF) genes [8]. Furthermore, YB-1 has been isolated as a major component of messenger ribonucleoprotein particles (mRNPs) that guide mRNA storage, for instance of GM-CSF [12] and renin [13], and is involved in translation processes [14-16]. The specific association of YB-1 with mRNA evidenced its regulatory role in mRNA processing in concert with splicing factors, such as SRp30c [17].

The plethora of functions fulfilled by YB-1 necessitates subcellular protein shuttling with high stringency. Specific protein domains, denoted nuclear export signals (NES) and nuclear localization signals (NLS), may coordinate such multifunctional shuttling and tasking [18]. Coordinated YB-1 protein shuttling has been characterized with in vitro cell models. Cell stress exerted by hyperthermia [19], cytotoxic agents [20], and ultraviolet irradiation [20] alters a predominant cytoplasmic YB-1 localization in unstressed cells to a nuclear preponderance. Cytokines IL-2 [21] and IFN-γ [6,22] are also reported to induce a similar nuclear shuttling. In vivo data have been mostly collected with cancer tissue. YB-1 has been detected in the cytoplasmic and/or nuclear compartments [23,24]. Nuclear YB-1 localization has been described as a negative prognostic marker for cancers of the breast [25], prostate [26], synovia [23,26], and lung [24]. Discussions have focused on the underlying mechanism(s) for poor cancer prognosis, e.g. the chemotherapy insensitivity of cells with high levels of nuclear YB-1 expression may be due to upregulated expression of multidrug resistance-1 (MDR-1 [10]) and the ABC transporter MRP2 [27].

Given the aforementioned tightly regulated subcellular distribution of YB-1 in inflammatory diseases and cancer, the present study was initiated to elaborate the underlying mechanisms that orchestrate YB-1 protein shuttling. Firstly, differences in subcellular targeting of fluorescently-tagged YB-1 domains was assessed in vitro using laser scanning microscopy [4]. Additionally, nuclear localization signals (NLS) that target domains of the protein, e.g. following endoproteolysis, to the nuclear compartment were fine-mapped. The functional relevance of a predefined carboxy-terminal fragment (CTF), that readily shuttles to the nuclear compartment, was unraveled, indicating an auto-inhibitory regulatory loop in gene transcription.

## Results

### Subcellular localization of YB-1 deletion constructs

**Figure 1:**
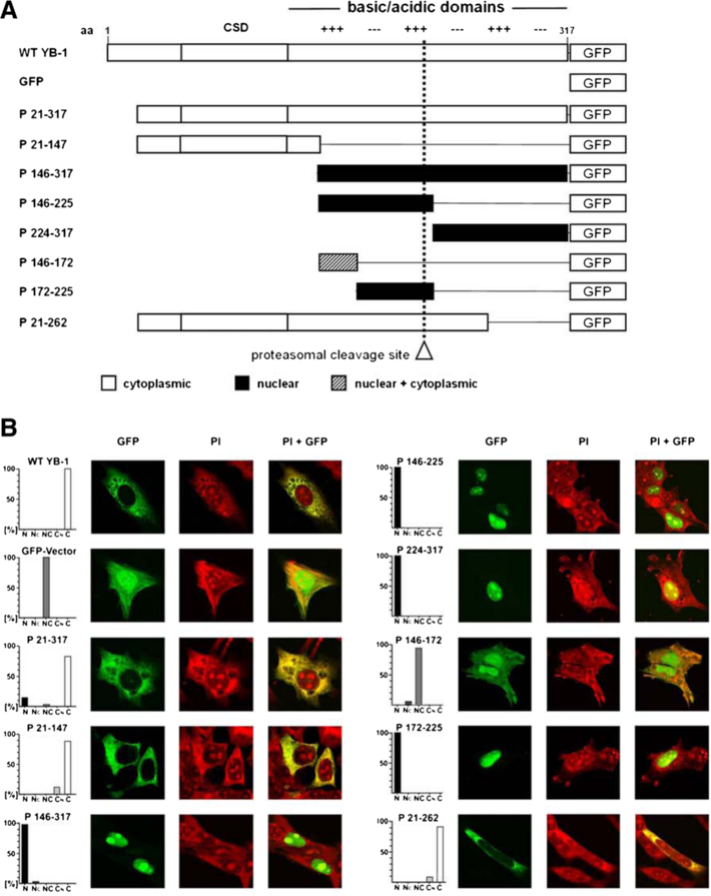
**Localization of YB-1 and deletion constructs of YB-1. A**. Schematic overview on GFP-tagged YB-1 (deletion) constructs. **B**. Localization of WT YB-1-GFP and YB-1 deletion constructs in proliferating RMC. Cells were transfected with expression vectors encoding for WT YB-1-GFP, YB-1 deletion constructs, or GFP. The subcellular localization was determined by confocal laser scanning microscopy, cell architecture was visualized by PI-staining. In the right column a merged overlay of PI staining and GFP fluorescence is shown. The percentages of cells with GFP-staining only in the nucleus (N), predominantly in the nucleus and weak in the cytoplasm (N_C_), in both compartments equally (NC), predominantly in the cytoplasm (C_N_), and only in the cytoplasm (C) are provided. 100 transfected cells were assessed for each plasmid.

Our starting hypothesis was that YB-1 protein fragments may be directed to different cellular compartments. Analyses of the subcellular distribution for full-length and truncated YB-1-GFP fusion proteins has been described in HeLa cells [4]. We first confirmed these results in our model system. Fusion proteins encompassing either the full-length YB-1 or various deletions, possessing a C-terminal green fluorescent protein tag, were introduced into rat mesangial cells (RMC; Figure 1A). Some constructs encode for proteins with truncations of the C-terminal domain (denoted basic/acidic (B/A) or charged zipper domain); depicted in Figure 1A. To preserve comparability with previous results, we chose to introduce the same expression constructs used by Jurchott et al. [4]. Of note, the protein fragments span aa 1–317 of the YB-1 protein (accession number J03827)[9] and not of YBX1 (accession number NM_004559), that has a disparate length of 324 amino acids due to a later annotation of the database.

Cells were grown without cell cycle synchronization in medium containing 10% FCS, as performed by Jurchott et al. [4]. For all immunofluorescence analyses, at least 100 transfected RMCs were assessed for their subcellular fluorescence distribution. Cells were grouped into five categories: (i.) exclusive nuclear (N), (ii.) exclusive cytoplasmic (C), (iii.) a pattern of uneven, predominantly nuclear (NC) or (iv.) predominantly cytoplasmic (CN) or (v.) even nuclear/cytoplasmic (NC) distribution. Laser scanning microscopy detected the full-length YB-1 protein fused to eGFP exclusively in the cytoplasm (Figure 1B). Transfection of the control eGFP plasmid resulted in staining of both the nuclear and cytoplasmic compartments (Figure 1B, second panel). The N-terminal YB-1 domains (aa 21–147) strictly localized to the cytoplasmic compartment, whereas the C-terminal domain aa 146–317 exhibited a nuclear localization (Figure 1B). Truncations within the YB-1 C-terminal domain, encoded by constructs P146-225 and P224-317, revealed that these are targeted to the nucleus, similarly to the fusion protein encompassing aa 172–225. The protein fragment P146-172 fused to eGFP was localized in both compartments. Of note, YB-1-eGFP fusion protein encoded by a longer construct covering aa 21–262 was exclusively detected in the cytoplasm, indicating that the nuclear localization signal(s) residing within aa 172–225 are not operative in a more extensive protein context that includes the N-terminal domains (Figure 1B). With the exception of the construct encoding for aa 21–262 all YB-1 deletion constructs behaved similarly, indicating that for the tested model systems there are no major differences with regard to YB-1 protein targeting.

### Fine-mapping of nuclear localization signals

**Figure 2:**
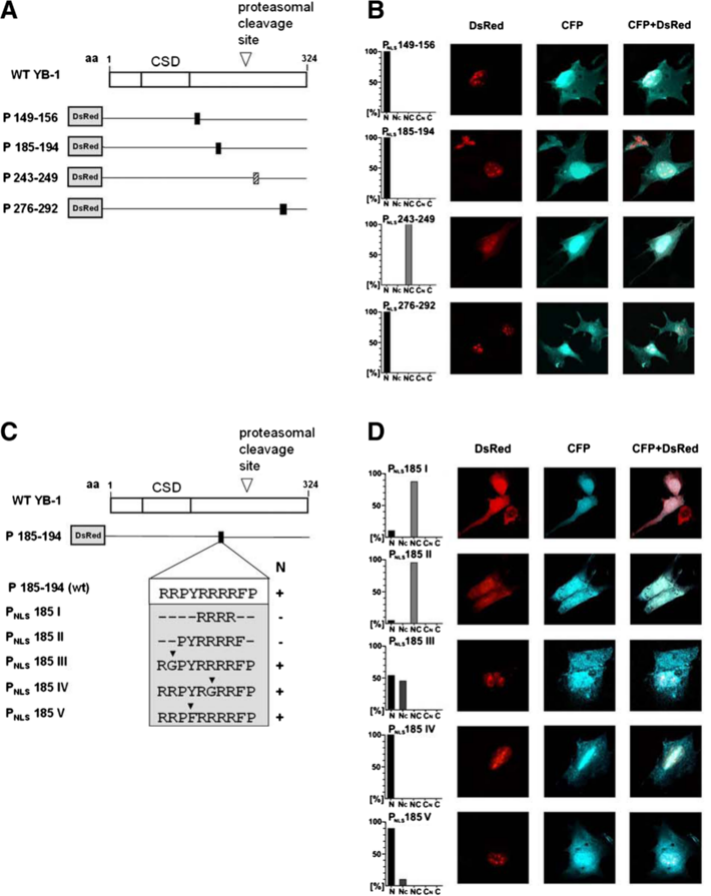
**Mapping the putative nuclear localization signals (NLS) of YB-1. A**. Diagram of DsRed-tagged abbreviated residues corresponding to putative NLS. The diagram summarizes the four putative NLS and their relative localization within the full-length YB-1 protein. **B**. Subcellular localization of putative NLS in RMC. Cells were transfected with expression vectors encoding for DsRed-tagged potential NLS of YB-1 and CFP, respectively. The subcellular localization was determined using confocal laser scanning microscopy. The cellular morphology was visualized by co-transfection with CFP, that enriches within the nucleus. The histograms provide quantification of the staining pattern. 100 transfected cells were assessed for each construct. **C**. Mutational analyses of the NLS-2 (P_NLS_185) motif. Diagram on DsRed-tagged mutated P_NLS_185 expression vectors generated to express the wild-type or mutated sequences (N: nuclear localization). **D**. Localization of the mutated P_NLS_185 expression proteins in RMC. Cells were transfected with DsRed-tagged expression vectors encoding for mutated P_NLS_185 and CFP, respectively. The histograms provide quantification of the staining pattern. 100 transfected cells were assessed for each construct.

To narrow down the nuclear localization signals within the YB-1 protein, a computer-based search for known NLS was performed using the NUCDISC program (http://psort.nibb.ac.jp; [28]). The search revealed four hits, all residing within the C-terminal basic/acidic domain, that are (i.) aa 149–156, (ii.) 185–194, (iii.) 243–249 and (iv.) 276–292. These motifs were tested in isolation by fusing them to a DsRed fluorescent tag at the N-terminus. The subcellular localization was determined following expression of the respective fluorescent proteins in RMCs (Figure 2A, B). To readily visualize the cellular compartments a plasmid encoding for cyan fluorescent protein (CFP) was co-introduced. CFP is predominantly detected within the nucleus at 552 - 627 nm. CFP was chosen as the DsRed tag fluorescence spectrum overlaps with that of propidium iodine, thus precluding this method for nuclear counterstaining.

The residues encompassing aa 149–156, aa 185–194 and aa 276–292 conferred an exclusive nuclear fluorescence pattern, whereas the aa 243–249 motif did not (Figure 2A, B). Since the motif at aa 149–156, denoted NLS-1, did not localize in the nucleus in the longer protein fragment encoded by P146-172 (Figure 1A, B), we therefore focused our attention on the motifs at residues aa 185–194 (NLS-2) and 276–292 (NLS-3) and evaluated their minimal composition for nuclear shuttling.

### Mutational analyses of the nuclear localization signals NLS-2 and −3

The NLS-2 at residue aa 185–194 has been described by Bader and Vogt in chicken YB-1 [29]. As a general rule, NLS are comprised of at least seven residues with a high content of basic amino acids [18]. Therefore we generated five different constructs by introducing mutations to narrow down the minimal requirement(s) for nuclear localization and specifically address the question whether the tyrosine residue at aa 188 is required for functionality (Figure 2C). With constructs P_NLS_ 185 I and P_NLS_ 185 II, an even cellular distribution of fusion proteins was observed (Figure 2D), indicating that the centrally located arginines alone are not sufficient for targeting. Exchange of either an arginine within the N-terminal portion (P_NLS_ 185 III) or a central arginine (P_NLS_ 185 IV) with the neutral amino acid glycine had a minimal effect on nuclear localization, when compared to the wild-type NLS-2 motif. Replacing the tyrosine with phenylalanine at aa 188 (P_NLS_ 185 V) also did not alter the nuclear localization, indicating that the minimal functional requirements of NLS-2 are independent of tyrosine 188 phosphorylation.

**Figure 3:**
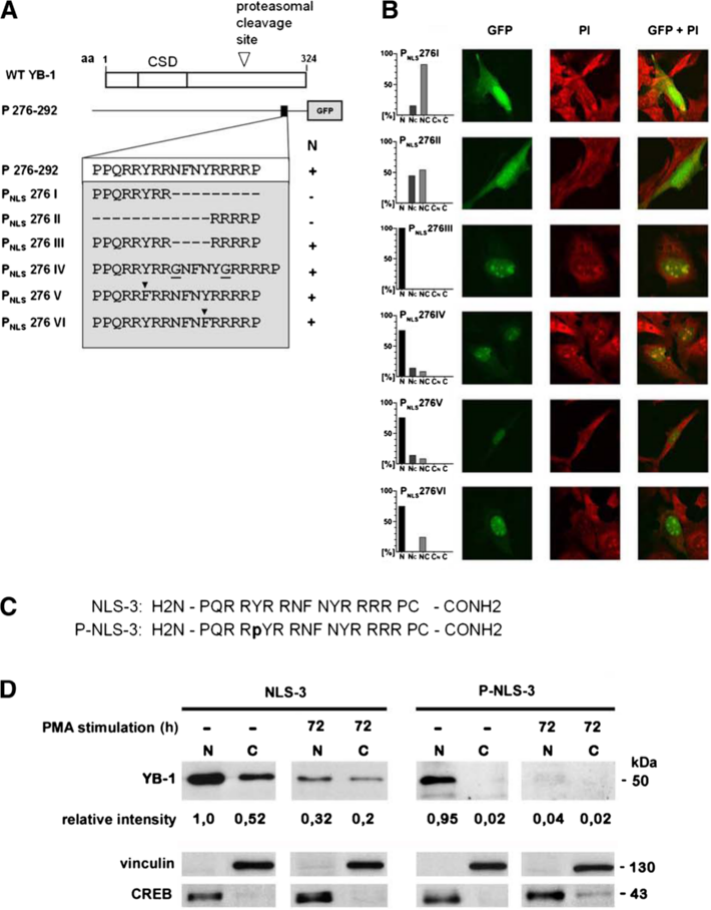
**Mutational analyses of NLS-3 (P**_**NLS **_**276) and immunoblotting of fractionated cell lysates. A**. Diagram of DsRed-tagged mutated P_NLS_276 expression vectors generated to express wild-type or mutated amino acid sequences (N: nuclear localization). **B**. RMC were transfected with EGF-tagged expression vectors encoding for the mutated P_NLS_276 sequence. The subcellular localization was assessed by laser scanning microscopy. The histograms provide quantification of the staining pattern. 100 transfected cells were assessed for each construct. **C**. Amino acid sequences of the peptides used for immunization and affinity purification. **D**. Nuclear YB-1 protein is phosphorylated at NLS-3 in non-differentiated monocytic, but not in differentiated THP-1 cells. Nuclear and cytoplasmic extracts were isolated from non-differentiated and differentiated THP-1 cells. Equal protein concentrations were loaded from each fraction. Immunoblotting was performed with the polyclonal antibodies generated against the unphosphorylated NLS-3 or tyrosine 281 phosphorylated sequence (P-NLS-3). Band intensities were quantified and normalized for nuclear CREB or cytoplasmic vinculin content. The cytoplasmic fraction has a 10× larger volume than the nuclear fraction.

Inspection of the domains at aa 276–292 (NLS-3) revealed a bipartite composition of this motif. Both “arms” of the motif, PPQRRYRR and RRRRP, exhibit characteristics of nuclear localization signals (Figure 3A). These motifs are separated by an interspersed linker comprised of four amino acids. Testing of the partite motifs in isolation, encoded by plasmids P_NLS_276 I and P_NLS_276 II, resulted in even cellular distribution of fluorescent protein. Deletion of the “linker” motif (NFNY; encoded by plasmid P_NLS_276 III) or extension of the “linker” by introduction of two additional glycine residues (encoded by plasmid P_NLS_276 IV) did not impair functionality of nuclear targeting. Furthermore, we tested whether substitution of either tyrosine residue affects nuclear localization. Tyrosines were mutated to phenylalanine in two separate constructs (P_NLS_276 V and VI), nevertheless, NLS-3 remained operative (Figure 3A, B).

### Phosphorylation at tyrosine 281 of NLS-3 correlates with nuclear YB-1 shuttling

The results obtained with the point-mutated NLS-3 motif (tyrosine exchanges) indicated that phenylalanine residues had no effect on the NLS-3 functionality in non-stimulated mesangial cells. Given that such results do not exclude phosphorylation of the tyrosine residues with subsequent alteration of functionality, we extended our analyses. As a model system we chose monocytic THP-1 cells, which express high levels of YB-1 and differentiate into adhering, amoeboid shaped macrophages following prolonged incubation with phorbol-12-myristate (PMA; 72 hours [30]). In accordance with previous work, a marked down-regulation of YB-1 expression was observed following PMA stimulation. It is known that PMA incubation affects intracellular signalling cascades and protein phosphorylation [30]. Western blot analyses were performed with two different polyclonal affinity-purified antibodies, prepared with unphosphorylated (NLS-3) and phosphorylated (P-NLS-3) peptides (Figure 3C). Phospho-specific antibody was affinity-purified following capture of the non-phospho-specific polyclonal immunoglobulins (flow-through following unphosphorylated peptide column incubation). This approach yielded highly specific immunoglobulin preparations suitable to determine the phosphorylation status, as confirmed in control blots with isolated peptides (not shown).

Fractionation of THP-1 cell lysates was performed and the phosphorylation status at NLS-3 assessed. Immunoblotting for cytoplasmic and nuclear marker proteins, vinculin and CREB, respectively, indicated successful fractionation. Undifferentiated THP-1 cells expressed YB-1 protein abundantly, with ~70% of nonphosphorylated YB-1 protein localizing to the nucleus (Figure 3D). Incubation of THP-1 cells with PMA resulted in decreased YB-1 protein expression (−80%) [30]. The YB-1 localization in differentiated THP-1 cells remained predominant nuclear (Figure 3D, left panel).

The same cell lysates were subjected to analyses with the phospho-specific antibody (Figure 3D, right panel). As a result, phosphorylation is only detected in the nuclear fraction of YB-1 in undifferentiated THP-1 cells. Cytoplasmic YB-1 was not detected, suggesting that cytoplasmic YB-1 is non-phosphorylated at NLS-3. In PMA-differentiated THP-1 cells, phosphorylation at tyrosine 281 was no longer detected. These results suggest that phosphorylation of tyrosine 281 in NLS-3 takes place and appears to correlate with nuclear protein shuttling (see also Additional file 1: Figure S1 and Additional file 2: Figure S2).

### Two putative cytoplasmic retention (CRS)/nuclear export signals (NES) exist within the YB-1 N-terminal domains

**Figure 4:**
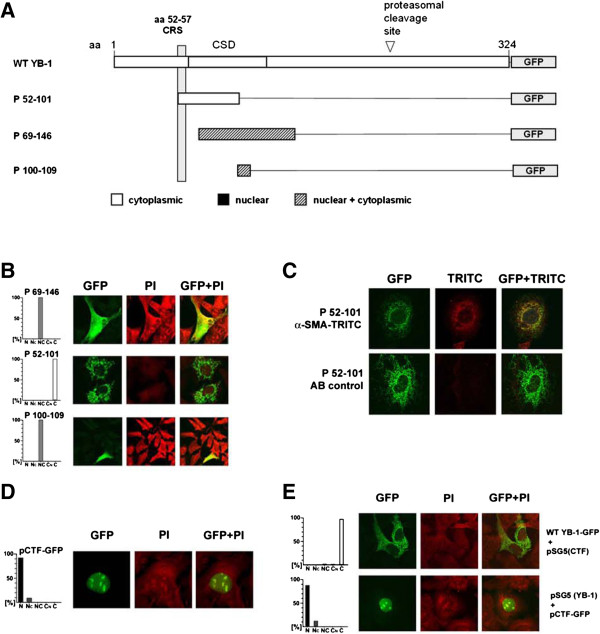
**Mapping of the potential nuclear export signals (NES) of YB-1 and subcellular localization of the carboxy-terminal YB-1 protein fragment (CTF). A**. Diagram on putative NES within the YB-1 protein. The composition of generated NES-constructs, denoted P1–57, P52–101, P52–146, and P69–146 with GFP-tags is provided. **B**. Localization of the tested constructs, denoted P69–146, P52–146, and P100–109, with GFP-tags in RMCs. The histograms provide quantification of the staining pattern. 100 transfected cells were assessed for each construct. **C**. Co-localization of P52–101 and α-smooth muscle actin in RMC. Cells were transfected with P52–101 (GFP-tagged) and after fixation, immuno-stained for α-smooth muscle actin (TRITC labelling). In the right column (GFP + TRITC) an overlay of TRITC and GFP staining is presented. **D**. Rat mesangial cells were transfected with an expression vector encoding for GFP-tagged CTF of YB-1 (pCTF). The subcellular localization was assessed using confocal laser scanning microscopy. The histogram provides quantification of the staining pattern. 100 transfected cells were counted. **E**. Localization of WT YB-1-GFP and CTF in co-transfected RMC. Cells were transfected with an expression vector encoding for WT YB-1-GFP and pSG-5(CTF), encoding for the untagged CTF. Furthermore, RMC were transfected with pCTF (GFP-tagged CTF) and the expression vector pSG5(YB-1) encoding for untagged YB-1. The subcellular localization was determined using confocal laser scanning microscopy. The histograms provide quantification of the staining pattern for 100 transfected cells.

YB-1 protein residues aa 21–147 and aa 21–262 were fused to eGFP at the C-terminus. The resultant hybrid proteins yielded exclusive cytoplasmic fluorescence patterns, indicating the presence of operative CRS or NES within the domains spanning aa 21–147 (Figure 1B). Next, four deletion constructs were designed that encoded for partially overlapping domains within the N-terminus: aa 1–57, aa 52–101, aa 52–146 and aa 69–146. The results indicate that only aa 52-101/eGFP showed an exclusive cytoplasmic localization (summarized in Figure 4A, B).

A computer-aided search for nuclear export signals (NetNES 1.1 Server, http://www.cbs.dtu.dk; [31]) identified one potential motif at aa 100–109 (**L**RS**V**GDGET**V**). Similar to classical NES, this motif contains three of the four properly spaced hydrophobic residues and is rich in the amino acids serine, glutamate, and aspartate. This motif was tested in isolation, fused to eGFP at the C-terminus, yielding an even cellular distribution (Figure 4B). Of note, fluorescent protein encoded by P52–101 was visualized in a punctuated pattern and pronounced in vicinity to the nuclear membranes. An interaction of YB-1 with actin myofibrils has been demonstrated by co-immunoprecipitation studies [32]. By double immunofluorescence staining we were able to demonstrate co-localization of actin fibers with ectopically expressed fluorescent P52-101 protein (Figure 4C).

### The carboxy-terminal YB-1 cleavage fragment is targeted to the nucleus

Sorokin et al. [33] described that YB-1 is endoproteolytically processed by the 20S proteasome between amino acids 119/220. The released N-terminal protein fragment was found to be transcriptionally active in the nucleus after thrombin stimulation of endothelial cells [34], whereas the carboxy-terminal fragment (CTF) has not been evaluated further. NLS-3 is situated within the 105 aa encompassing the CTF , suggesting that the CTF may also be targeted to the nucleus. The subcellular targeting of the CTF was analyzed by means of a construct encoding the CTF (aa 220–324) fused to eGFP at the C-terminus. This fluorescent protein was exclusively localized in the nucleus, with a pronounced speckled pattern (Figure 4D). We next asked whether over-expression of CTF affects the subcellular localization of full-length YB-1 protein, given that it may act as a decoy protein for the dimerization motif. The cellular content of untagged CTF was increased by means of expression vector pSG5(CTF). The co-introduced fluorescent full-length YB-1 protein was detected by laser scanning microscopy. In a reciprocal approach, full-length YB-1 protein levels were increased using the expression vector pSG5(YB-1) and the CTF fluorescent protein detected. Under both conditions the described compartmentalization of the proteins remained unaltered (Figure 4E).

### YB-1 proteolysis and subcellular targeting of domains following genotoxic stress

**Figure 5:**
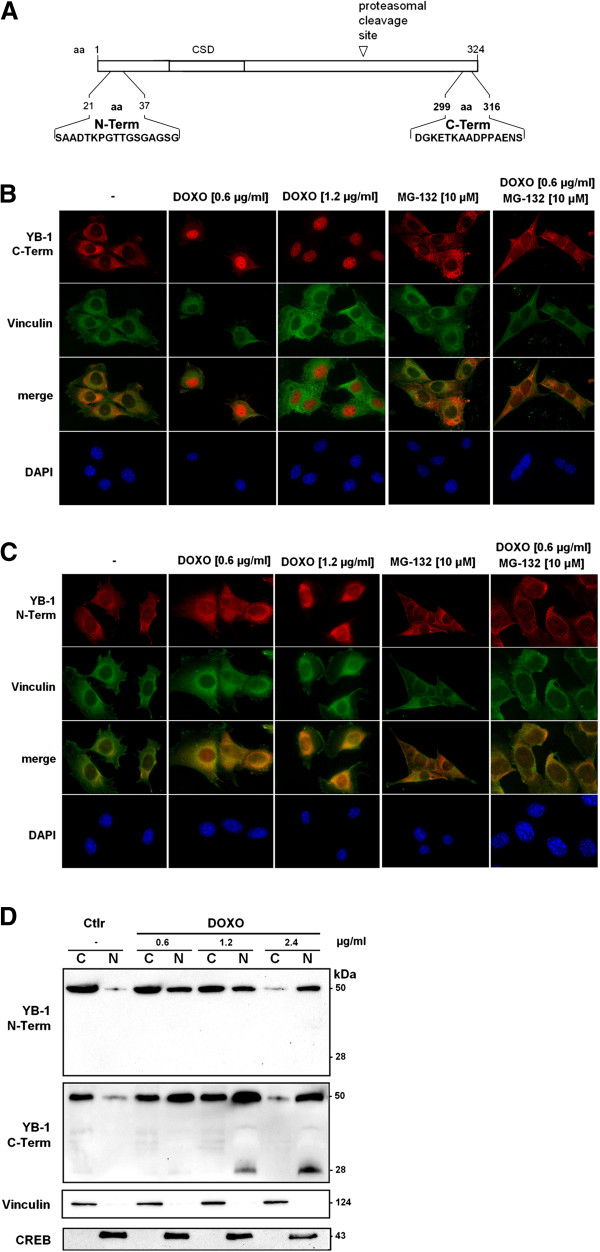
**Subcellular localization of YB-1 protein fragments following genotoxic stress in the absence and presence of proteasome inhibitor. A**. Schematic of YB-1 protein with its centrally localized cold shock domain. Polyclonal peptide-derived affinity-purified antibodies were generated against two epitopes localized either in the N- or C-terminus. **B**. Distribution of endogenous YB-1 protein was assessed by immunofluorescence microscopy in rat mesangial cells following immunodetection with the anti-YB-1 antiserum directed against the C-terminus (primary antibody). Murine anti-vinculin was used to visualize the cell structure. Fluorescently labelled secondary antibodies anti-rabbit IgG(Fab)-Cy3 and anti-mouse IgG(Fab)-FITC were used for detection. Nuclei were visualized by DAPI staining. Rat mesangial cells were incubated for 16 h with doxorubicin at increasing concentrations (0.6 and 1.2 μg/ml) in the absence or presence of proteasome inhibitor MG-132 (10 μmol/l). Images were taken at ×63 magnification. **C**. Distribution of endogenous YB-1 protein was assessed by immunofluorescence microscopy in rat mesangial cells according to the protocol outlined in **A** with polyclonal YB-1 antiserum directed against the N-terminus (N-Term, primary antibody). **D**. Immunoblotting of fractionated cell lysates from rat mesangial cells exposed to doxorubicin at increasing concentrations (0.6 , 1.2, and 2.4 μg/ml). Cytoplasmic and nuclear proteins were separated and purity ascertained by detection of vinculin and CREB.

The nuclear shuttling of YB-1 has been described under conditions of cellular stress. To address the question, whether proteolytic processing of YB-1 and nuclear shuttling of the resulting protein fragments takes place cytotoxic stress experiments were designed. Doxorubicin, a common drug used to treat cancers of the bladder, breast, lung, or ovary, was added to the culture medium of MCF-7 breast cancer and rat mesangial cells in the absence and presence of the proteasome inhibitor MG-132. The subcellular distribution of endogenous YB-1 protein was assessed using affinity-purified polyclonal antibodies targeting epitopes within the protein N- and C-terminus (Figure 5A). Immunofluorescence microscopy revealed that antibody preparations were specific for YB-1, (Additional file 3: Figure S3). In unchallenged cells, the C-terminal antibody detected YB-1 predominantly within the cytoplasm. A concentration-dependent shift to the nucleus was visualized following doxorubicin incubation (DOXO, 0.6 and 1.2 μg/ml; Figure 5B). In order to assess whether cleavage of YB-1 is a prerequisite for nuclear translocation, proteasome inhibitor MG-132 (10 μM) was added to the cell culture medium and the cells challenged with doxorubicin. In cells preincubated with MG-132, most YB-1 protein remained cytoplasmic with only a minor fraction shuttling to the nucleus (Figure 5B). From this observation it may be concluded that proteasomal cleavage appears to be a prerequisite for nuclear translocation; although additional “activation” mechanisms may be operative. Next, the antibody specific for an epitope within the protein N-terminus was utilized. In unchallenged cells, the N-terminal antibody detected YB-1 predominantly within the cytoplasm, similar to the results obtained with the C-terminal antibody. However, following genotoxic stress (Figure 5C), some YB-1 protein was still detected perinuclear. Preincubation with MG-132 yielded a predominantly cytoplasmic fluorescence pattern. The results suggest that cell stress-dependent protein cleavage is followed by nuclear shuttling of the protein C-terminal domain. To confirm that such a cleavage event occurs, cell fractionation was performed with rat mesangial cells exposed to increasing concentrations of doxorubicin (0.6, 1.2, and 2.4 μg/ml), followed by immunoblotting with the aforementioned antibodies (Figure 5D and Additional file 2: Figure S2). As a result, a concentration-dependent nuclear accumulation of full-length YB-1 was detected with both antibodies. In addition, a protein fragment with a relative molecular weight ~28 kDa was detected following doxorubicin exposure. This fragment is found exclusively in the nuclear fractions using the antibody recognizing the C-terminal epitope and is phosphorylated at NLS-3 (Additional file 2: Figure S2). Similar results were obtained using MCF-7 breast cancer cells (Additional file 4: Figure S4A/B). Again, a concentration-dependent shift of YB-1 to the nuclear compartment was visualized following doxorubicin incubation, a proteasome-dependent cleavage was evidenced by the same subcellular alterations as described for rat mesangial cells. Cell viability was assessed by trypan blue exclusion in control experiments with increasing concentrations of doxorubicin (Additional file 4: Figure S4C).

### The CTF influences the transcriptional activity of full-length YB-1

**Figure 6:**
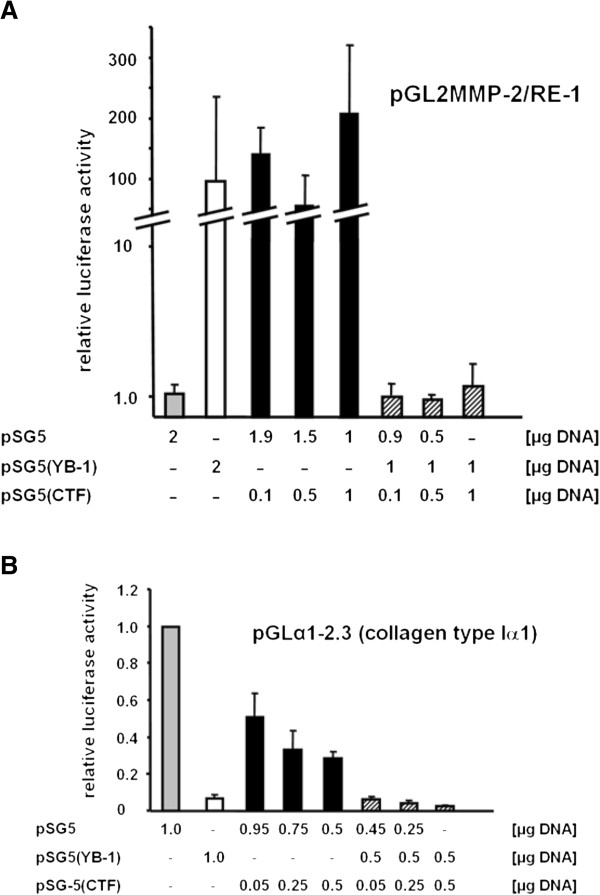
**The carboxy-terminal YB-1 protein fragment (CTF) regulates transcriptional activity. A**. Transcriptional activity of CTF on the MMP2 promotor construct pGL2MMP-2/RE-1. Rat mesangial cells were transfected with equal amounts of DNA (as indicated). After 48 hours cell lysates were prepared and luciferase assays were performed. All experiments were performed in triplicates and confirmed in three independent experiments. **B**. Transcriptional activity of the CTF on the Col1a1 promotor construct pGLα1-2.3. Rat mesangial cells were transfected with the indicated expression vectors and incubated for 48 hours. Luciferase assays were performed thereafter using cell lysates. All experiments were performed in triplicates and confirmed in three independent experiments.

In the following, we tested whether ectopically expressed CTF interferes with gene transcription orchestrated by overexpressed full-length YB-1, e.g. target gene MMP-2. Rat mesangial cells were transfected with the reporter construct pGL2MMP-2/RE-1 that harbours a YB-1 responsive enhancer element derived from the rat MMP-2 promoter [35,36]. The experimental set-up included co-transfections with empty expression vector pSG5, pSG5(YB-1), pSG5(CTF), or the combination of the latter. As described before, full-length YB-1 overexpression enhanced the transcriptional activity of the MMP-2 promoter more than 100-fold under the chosen conditions ([11], Figure 6A). A similar induction of gene transcription was observed with ectopically expressed CTF, ranging between 100- and 200-fold even at low doses of plasmid DNA. When both expression plasmids, encoding full-length YB-1 or CTF, were co-transfected, the transcriptional activity was markedly repressed. Quantification revealed luciferase values at background levels (Figure 6A). The abrogation of full-length YB-1 transcriptional activity was determined with increasing concentrations of CTF expression plasmid. Thus, the CTF may functionally interfere with the *trans*-stimulatory effect of full-length YB-1 on target gene expression, whereas the CTF alone is capable of *trans*-activating gene transcription to a comparable extent as full-length YB-1 protein.

A *trans*-repressive effect of YB-1 on gene transcription, e.g. of the col1α1 promoter, has also been described [37]. We next wished to evaluate whether the CTF alone may fulfill such a repressive effect. Rat mesangial cells were transfected with the well characterized promoter reporter construct pGLα1-2.3 [37]. Ectopic overexpression of full-length YB-1 led to a suppression of transcriptional promoter activity by more than 90%. Ectopic overexpression of CTF alone resulted in a similar reduction of promoter activity by ~70% (Figure 6B). Co-expression of both, full-length YB-1 protein and CTF, did not interfere with the repressive effect.

## Discussion

Our first quest was to determine the subcellular localization of full-length YB-1 protein as well as a series of truncated protein fragments (Figure 1). We confirmed in resting cells that full length YB-1 is primarily cytoplasmic. Construct encoding aa 21 to 262 yielded an equal fluorescent signal in the cytoplasm and nucleus, whereas it was exclusively cytoplasmic the previous study [4]. For the other constructs, no differences regarding subcellular targeting of YB-1 protein was detected in vitro.

Next we mapped the functional motifs and subdomains within the YB-1 protein that either confer nuclear shuttling/retention or cytoplasmic localization/retention. Analogous to the report by Bader et al. [29] we detected functionality of the NLS at aa 185–194 (referred to here as NLS-2) in rat cells. In addition, two novel nuclear localization motifs located at aa 149–155 (NLS-1) and aa 276–292 (NLS-3) were mapped. Inspection of motif NLS-3 immediately suggested a bipartite composition. Mutational analyses confirmed that both parts are indeed required for functionality, at the same time alterations of the spatial organization of the two halves did not impair nuclear shuttling of a fluorescent fusion protein. Both NLS sequence motifs (NLS-2 and −3) contain tyrosine residues that are potential sites for phosphorylation, e.g. to regulate functionality of the NLS. To determine whether phosphorylation take place, we generated polyclonal peptide-based antibodies against the non-phosphorylated NLS-3 domain and the same antigen phosphorylated at tyrosine 281. The results indicate that most of the YB-1 protein phosphorylated at tyrosine 281 within NLS-3 appears to be in the nucleus, whereas the cytoplasmic YB-1 protein appears unphosphorylated at this residue (Figure 3 and Additional file 2: Figure S2). These results indicate that phosphorylation at tyrosine 281 is accompanied by nearly exclusive nuclear localization of full-length YB-1, suggesting that the phosphorylated NLS-3 may act dominantly on the subcellular localization of Y-box protein. However, the data also show that this phosphorylation is not essential for nuclear localization. In addition, the data suggest that the localization of YB-1 may be highly regulated after cell stimulation. Stenina et al. [34] and Sorokin et al. [33] observed a nuclear localization of the truncated YB-1 protein containing N-terminal portions of the protein in stimulated cells. For the amino-terminal domains of YB-1, a strict cytoplasmic localization was observed when the expressed YB-1 deletion/GFP-fusion proteins harbour aa 52–101. In an attempt to define nuclear export signal-containing domains further constructs were designed that mapped to diverse regions of the protein N-terminus (see Figure 6). However all of these “truncated” constructs lost their subcellular specification.

In summary, we could identify 3 different NLS, but no NES within the YB-1 protein. The existence of three different NLS within a protein underscores a careful regulation of its subcellular localization. The localization of phosphorylated NLS-3 within the nucleus of unstimulated cells raises many questions regarding its regulation. However, further scrutiny of the other NLSs shows that they also contain tyrosine residues, several for which the phosphorylation has already been reported (PhosphoSitePlus). Thus, the regulation of tyrosine phosphorylation and its role in the nuclear localization of YB-1 is an area that requires further study. Previous reports by Bader et al. have identified aa 137–183 in participating as a multimerization domain of chicken YB-1 [29]. YB-1 multimerized in the presence and absence of DNA and RNA templates [38]. Therefore, it is possible that in the aggregates of multimerized YB-1, the region harboring the NLS-1 is hidden and thus no longer accessible to proteins involved in nuclear import.

Information on the subcellular localization of YB-1 protein in healthy tissue or in inflammatory diseases is scarce. In healthy kidney tissue, YB-1 was almost exclusively detected in the nucleus of glomerular and tubulointerstitial resident cells [39]. Following the induction of mesangioproliferative disease, a temporally and spatially coordinated up-regulation of YB-1 was detected in the cytoplasm of mesangial cells [39]. Such a tight regulation of the subcellular protein specification is one aspect that must be fulfilled to explain its involvement in pleiotropic functions, ranging from gene transcription to pre-mRNA splicing, mRNA translation, secretion, and receptor interaction [40,41].

In the studies by Sorokin et al. [33] the focus was placed on the N-terminal fragment. Somewhat surprisingly, we did not detect this fragment with our N-terminal antibody after stress induction (Figure 5D), however we clearly detect the CTF, which may suggest a differential processing of YB-1 that is signal-dependent. Our inability to detect the N-terminal fragment of YB-1 suggests that it is either post-translational modified, thus masking the epitope, or degraded. While there are no reported phosphorylation sites within the epitope (aa 10–22), there are multiple lysine residues that are sites of ubiquitinylation (PhosphoSitePlus^®^[42]). Additionally, YB-1 associates via its N-terminus with FBX33, a component of an SCF E3–ubiquitin ligase; an interaction that targets YB-1 for proteasomal degradation [43]. However, the issue of whether proteolytic processing of YB-1 occurs is still disputed [44,45].

We extended the functional analyses by creating an expression plasmid encoding for the CTF only. The effect of ectopically expressed CTF on gene transcription from enhancer and silencer elements, respectively, of YB-1 target genes MMP-2 and collagen type I [11,37] were analyzed. In rat mesangial cells ectopic overexpression of the CTF resulted in increased transcriptional activity of the MMP-2 response element-1, as was shown before for full-length YB-1 (Figure 6). Notably, co-expression of CTF and full-length YB-1 resulted in a loss of transcriptional *trans*-activation of the same element. Thus, the carboxy-terminal YB-1 protein fragment aa 220–324, lacking the described DNA- or RNA-binding cold shock domain [46], acts dominant-negatively on full-length YB-1-dependent gene transcription. The CTF, which is composed of basic/acidic repeats, is generally thought to mediate protein-protein interactions [1]. However, the B/A motifs from the C-terminus of Y-box proteins have also been shown to have nucleic acid-binding activity [46,47]. Thus, a DNA binding mechanism would be an obvious explanation, however other possibilities could be envisioned (e.g. a decoy function that influences protein-protein interactions to promote or inhibit complex formation or YB-1 oligomerization). Further investigations will be required to elucidate this mechanism of action.

**Figure 7:**
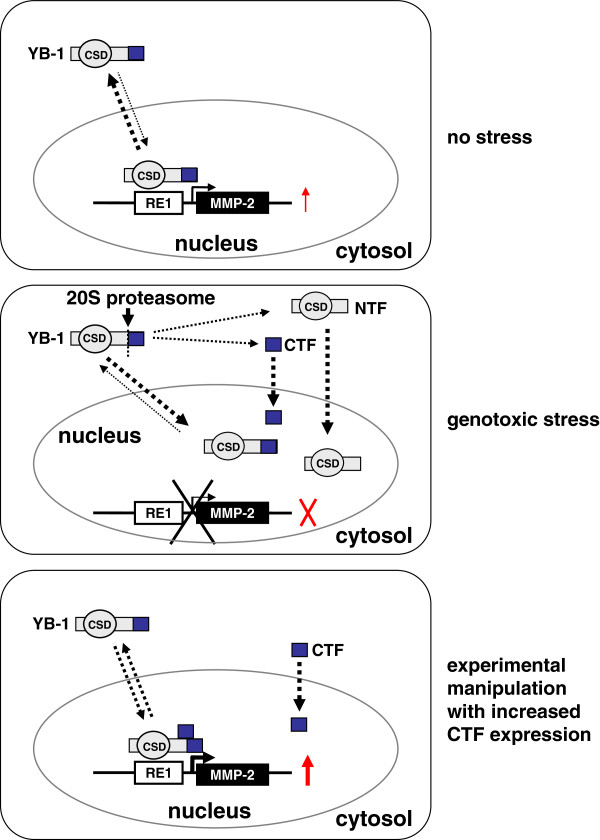
**Model of the functional activities for the C-terminal fragment (CTF) of YB-1.** In non-stressed cells full-length YB-1 protein is predominantly cytoplasmic and may shuttle between the nucleus and cytoplasm. In the nucleus, full-length YB-1 binds to the RE-1 element within the MMP-2 promotor and *trans*-activates gene transcription. Following genotoxic stress, full-length YB-1 is predominantly localized in the nuclear compartment. In addition, cleavage of full-length YB-1 protein by the 20S proteasome takes place; whether this occurs in the cytoplasm or nucleus has not been investigated. The cleaved C-terminal fragment (CTF) also resides within the nuclear compartment. Co-localization of full-length YB-1 and CTF in the nuclear compartment results in loss of transcriptional *trans*-regulation of the MMP-2 promoter. The MMP-2 promoter is transcriptionally activated by ectopically expressing CTF, that readily shuttles to the nucleus.

A hypothetical model for the functionality of the CTF was set up, emphasizing the need for cooperative protein interactions to direct gene transcription and also the novel regulatory ramifications with the CTF acting on gene transcription. In normal, unstressed cells full-length YB-1 is primarily cytoplasmatic (Figure 7). In the case of genotoxic stress, a subset of the full-length YB-1 protein is cleaved by the 20S proteasome. Both the full-length protein and the cleavage products localize to the nucleus, resulting in a loss of transcriptional activity at the MMP-2 promoter. This effect may be dependent on the half lives of the distinct protein fragments (Figure 7). It will be of interest to see whether other functions of YB-1, such as mRNA binding and translation processes, are also regulated by the CTF. Of note there was a *trans*-repressive effect of the CTF on the collagen type I promoter element, that was not subjected to interference with wild-type protein activities. Thus it appears that the activating and repressive activities of YB-1 on gene transcription may involve mechanisms that reflect a requirement for different levels of YB-1 oligomerization and/or perhaps different binding partners.

## Conclusions

The full spectrum of signals that induce either the phosphorylation and/or proteolytic processing of YB-1 still remains to be determined. It is interesting to speculate to what extent these processes may contribute to pathology, particularly as enhanced levels of nuclear YB-1 are often associated with a poor prognosis in cancer [45,48,49]. Proteasome inhibitors have been introduced into therapeutical regimens of hematological disorders like multipe myeloma. Of note, experimental evidence indicates that proteasomal inhibitors like bortezomib may exert profound antiinflammatory activities in kidney diseases, like lupus nephritis [50] and necrotizing glomerulonephritis [51]. Future studies will address the important question of whether the prevention of YB-1 cleavage via proteasomal inhibition contributes to these anti-inflammatory activities.

## Methods

### Cell culture

Rat mesangial cells were established as previously described [52,53]. Human monocytic THP-1 as well as human breast adenocarcinoma MCF-7 (Michigan Cancer Foundation-7) cells were obtained from the ATCC. RMCs and THP-1 cells were grown in RPMI 1640 medium supplemented with 10% fetal calf serum, 2 mM L-glutamin, 100 μg/ml of streptomycin and 100 U/ml penicillin at 37°C in humidified 5% CO_2_. MCF-7 cells were grown in DMEM medium supplemented as described above.

### Plasmids

Plasmids encoding for the WT YB-1 fusion protein and the GFP-tagged deletion constructs were obtained from K. Jürchott (Max-Delbrück Center, Berlin, Germany). For characterization of the NLS and NES, DNA fragments of the YB-1 sequence (gene AC J03827) were cloned into the vector pDsRed2-C1 or pEGFP-N1 (BD Biosciences Clontech, Heidelberg, Germany). The fusion proteins were tagged with DsRed2 at the N-terminus or EGFP at the C-terminus, respectively. Inserts were generated by PCR using the full-length WT YB-1 vector as template and primers as described in Additional file 5: Table S1, and digested with EcoRI/BamHI or BglII/EcoRI. For the generation of the small deletion constructs and mutational analyses annealed oligonucleotides (Invitrogen, Karlsruhe, Germany), as described in Additional file 6: Tables S2 and Additional file 7: Table S3, were ligated into pDsRed2-C1 or pEGFP-N1 vectors, respectively. All nucleotide sequences were verified by automated sequencing.

### Transient transfection and laser scanning microscopy

RMC were grown to 60-80% confluency on coverslips in 6-well culture plates and transiently transfected with Fugene® 6 according to the manufacturers instructions (Roche, Basel, Switzerland). Briefly, 2 μg plasmid DNA and 6 μl Fugene solution were gently mixed in 80 μl serum-free RPMI 1640 medium and incubated for 1 hour. RMC transfected with pDSRed2 derived vectors (1 μg) were cotransfected with 1 μg of pCFP vector (BD Biosciences Clontech, Heidelberg, Germany). The transfected cells were washed twice with PBS and then fixed with 4% paraformaldehyde/PBS for 30 minutes. In transfection studies with GFP-tagged vectors, nuclear staining was achieved by adding propidium iodine in a concentration of 5 μg/ml for 10 min at room temperature. Cells were mounted with immuno-mount (Shandon, Pittsburgh, USA) and analyzed by laser scanning microscopy (Axiovert 100 M confocal laser scanning microscope, Carl Zeiss, Oberkochen, Germany), using a dual parameter setup and dual wave-length excitation at 488 nm and 543 nm for detection of GFP/PI fluorescence or 458 nm and 543 nm for DsRed/CFP fluorescence. All transfection experiments were performed at least three times.

### Luciferase assays

For luciferase activity measurements, RMC were transfected with pSG5, pSG5(YB-1), and/or pSG5(CTF) expression vectors together with the promoter constructs pGL2MMP-2/RE-1 in 6 or 12 well plates and incubated for 48 hours. After 48 hours, cells were harvested in 100 μl reporter lysis buffer and luciferase assays were performed with 20 μl of lysates using the Luciferase assay system (Promega, Madison, WI, USA). All assays were performed in triplicate.

### THP-1 differentiation

THP-1 cells were differentiated into adherent macrophage-like cells by incubation with phorbol-12-myristate for 72 hours (PMA, 100 nM; Sigma-Aldrich, Seelze, Germany). Nuclear and cytoplasmic cell extracts were prepared as previously described [11]. Protein concentrations were determined sing the Bio-Rad protein assay and bovine serum albumin as a standard. Proteins were subjected to SDS-PAGE, transferred to nitrocellulose, and detected with suitable polyclonal antibodies to NLS-3, or P-NLS-3 (Eurogentech, Köln, Germany), vinculin, or CREB. Protein bands were visualized using ECL (Amersham Biosciences, Piscataway, NJ, USA). Band intensities were quantified using the Scion Image software. The YB-1 protein content in cells without PMA-treatment was set to one.

### Antibodies

Two peptide-derived rabbit polyclonal anti-YB-1 sera, recognizing epitopes aa 21–37 and 306–321 of YB-1, were generated as previously described [39,40,54,55]. Sera were used at a dilution of 1:100 for immunofluorescence (IF) and 1:1000 for Western blotting (WB). A monoclonal antibody against Vinculin was purchased from (Fitzgerald Industries, Acton, Massachusetts, USA) and used at 1:100 for IF and 1:1000 for WB. A monoclonal antibody against CREB was purchased from (Cell Signaling, Danvers, MA, USA) and used at 1:1000 for WB. Horse radish peroxidise-linked anti-rabbit and -mouse antibodies (SouthernBiotech, Birmingham, Alabama , USA) for WB (dilutions 1:5000 to 1:10,000). Cy3-labeled anti-rabbit antibody (Sigma-Aldrich, Seelze, Germany) and FITC-labeled anti-mouse antibody (Dako Deutschland GmbH, Hamburg, Germany) were used for IF**.**

### Cell viability

A trypan blue (Sigma-Aldrich, Seelze, Germany) exclusion assay was performed as described [52].

### Cytoplasm and nuclear fractionation

RMC and MCF-7 cells were washed twice with PBS and lysed in Nuclear Extraction Buffer A (10 mM Hepes, 10 mM KCl, 0.1 mM EDTA) containing complete protease inhibitor cocktail (Roche, Basel, Switzerland) at 4°C for 15 min and centrifuged at 15,000 × g for 3 min at 4°C. Supernatants containing cytoplasmic proteins were collected. Pellets resuspended in Nuclear Extraction Buffer A and centrifuged at 15,000 × g for 3 min at 4°C. Supernatants were decanted. Steps were repeated two more times. Pellets resuspended in Nuclear Extraction Buffer B (20 mM Hepes, 0.4 M NaCl, 1 mM EDTA, 10% Glycerol) containing complete protease inhibitor cocktail (Roche) at 4°C for 20 min and centrifuged at 25,000 × g for 5 min at 4°C. Supernatants containing nuclear proteins were collected and protein concentrations of the different fractions determined by BioRad protein assay (BioRad, Munich, Germany) with BSA as a standard.

### Western blotting

Denatured protein samples were separated by electrophoresis in 10% SDS-PAGE, transferred onto nitrocellulose membranes, blocked with 5% milk in PBS-tween (PBST), washed with PBST, and incubated with primary antibodies (described above) diluted in PBST overnight at 4°C. Primary antibodies were detected using HRP-conjugated secondary antibodies (described above) diluted in PBST for two hours at room temperature.

### Immunofluorescence microscopy

MCF-7 and RMC cells were grown on glass coverslips. Twenty-four hours later, the cells were treated with different concentration of doxorubicin for fourteen hours. After incubation with doxorubicin, the cells were washed twice with PBS to remove non-adherent cells and fixed with 4% paraformaldehyde in PBS. Two different peptide-derived rabbit polyclonal anti-YB-1 antibodies and mouse anti-viniculin were used (1:100). Secondary antibodies Cy3-labeled anti-rabbit (Sigma-Aldrich, Seelze, Germany), and FITC-labeled anti-mouse (Dako Deutschland GmbH, Hamburg, Deutschland) were diluted 1:300 . Nuclei were counterstained with DAPI (Invitrogen, Karlsruhe, Germany). Cells were mounted with fluorescence mounting medium (Dako Deutschland GmbH, Hamburg, Germany) and analyzed using a fluorescence microscope (DM6000 B; Leica Microsystems GmbH, Darmstadt, Germany) equipped with a CCD Camera (DFC340 FX; Leica Microsystems GmbH, Darmstadt, Germany) and a 63/1.4 objective. Separate images were taken and later merged using ImageJ™ software.

## Abbreviations

CFP: Cyan fluorescent protein; CRS: Cytoplasmic retention signal; CSD: Cold shock domain; CSP: Cold shock protein; CTF: Carboxy-terminal fragment; DMEM: Dulbecco’s Modified Eagles Medium; DOXO: Doxorubicin; eGFP: Enhanced green fluorescent protein; FITC: Fluorescein isothiocyanate; GM-CSF: Granulocyte macrophage-colony stimulating factor; IF: Immune fluorescence; IFNγ: Interferon gamma; IL-2: Interleukin 2; MCF-7: Michigan Cancer Foundation-7; MDR-1: Multidrug resistance 1; MMP-2: Matrix metalloproteinase 2; mRNPs: Messenger ribonucleoprotein particles; MRP2: Multidrug resistance protein 2; NES: Nuclear export signals; NLS: Nuclear localization signals; PBS: Phosphate buffered saline; PBST: Phosphate buffered saline tween; RMC: Rat mesangial cells; SDS-PAGE: Sodium dodecylsulfate-polyacrylamide gel electrophoresis; WB: Western blotting; YB-1: Y-box protein-1.

## Competing interests

The authors declare no competing financial interests.

## Authors’ contributions

C.R.C.vR., F.G.S. designed and performed experiments, analyzed and interpreted results, and wrote the manuscript; S.B., V.A.K., S.M., S.D., U.R. performed experiments and analyzed data; H-D.R. designed and contributed vital reagents; S.E.D., S.D., H.W. designed experiments, interpreted results, and wrote the manuscript; T.F. designed contributed vital reagents, analyzed and interpreted results; J.A.L. designed and performed experiments, analyzed and interpreted results, and wrote the manuscript; and P.R.M. directed the study, designed experiments, supervised the work, interpreted results, and edited the manuscript. All authors read and approved the final manuscript.

## Supplementary Material

Additional file 1: Figure S1 Antibody specificity testing with and without calf intestinal alkaline phosphatase treatment. Rat mesangial cells were left untreated (-) or treated (+) with the protein tyrosine phosphatase inhibitor pervanadate (PV) to maximize phosphorylation. Cell lysates were loaded in duplicate, separated by SDS-PAGE, and transferred onto membranes. The membrane was cut and blocked. In addition, one membrane was incubated in alkaline phosphate buffer with 1U calf intestinal alkaline phosphatase (CIP)/ μg protein for 60 minutes (+CIP). The membranes were the incubated with primary antibody as indicated and visualized using ECL detection. As seen, CIP treatment completely ablates the signal detected using the pNLS3 antibody demonstrating its phospho-specificity. The pan-phosphotyrosine antibody [4G10] shows that not all tyrosine phosphorylation has been removed. The YB-1 and β-tubulin signals are comparable. The position of the protein standards and the relative molecular weight (MW) in kiloDaltons (kDa) are indicated. Click here for file

Additional file 2: Figure S2 Subcellular localization of YB-1 protein fragments following genotoxic stress. Immunoblotting of fractionated cell lysates from rat mesangial cells exposed to doxorubicin for 14 h at increasing concentrations (0.6 , 1.2, and 2.4 μg/ml). Cytoplasmic and nuclear proteins were separated and purity ascertained by detection of vinculin and CREB. Additionally, blotting with the pNLS3 antibody shows that the phosphorylated C-terminal fragment (p28) is found exclusively in the nuclear fraction. Click here for file

Additional file 3: Figure S3 Antibody specificity testing with preincubation of immunization peptides in MCF-7 cells. Distribution of endogenous YB-1 protein was assessed by immunofluorescence microscopy in MCF-7 cells with a peptide-derived affinity purified polyclonal YB-1 antiserum directed against the N-terminus (primary antibody). Upper left panel: untreated N-terminal antibody. Upper middle panel: antibody mixed with 0.1 μg/ml of immunizing peptide (YB-1 amino acids 21 to 37: SAADTKPGTTGSGAGSG). Upper right panel: antibody mixed with with 1 μg/ml of immunizing peptide. Middle panels: Murine anti-vinculin antibody was utilized to visualize the cell structure. Lower panels: Nuclei were visualized with DAPI. Images were taken at ×63 magnification. Click here for file

Additional file 4: Figure S4 Subcellular localization of YB-1 protein fragments following genotoxic stress in the absence and presence of proteasomal inhibitor in MCF-7 breast cancer cells. 1 2. **A**. Distribution of endogenous YB-1 protein was assessed by immunofluorescence microscopy in MCF-7 cells following immunodetection with the anti-YB-1 antiserum directed against the C-terminus (primary antibody). Murine anti-vinculin antibody was utilized to visualize cell structures. Fluorescence labelled secondary antibodies consisted of anti-rabbit IgG(Fab)-Cy3 and anti-mouse IgG(Fab)-FITC. Nuclei were visualized by DAPI staining. MCF-7 cells were incubated for 16 h with doxorubicin at increasing concentrations (0.6 and 1.2 μg/ml) in the absence or presence of proteasome inhibitor MG-132 (7.5 and 10 μmol/l). Images were taken at ×63 magnification. **B**. Distribution of endogenous YB-1 protein was assessed by immunofluorescence microscopy in MCF-7 cells according to the protocol outlined in **A** with polyclonal YB-1 antiserum directed against the N-terminus (N-Term, primary antibody). **C**. Cytotoxicity assay with increasing concentrations of doxorubicin. Rat mesangial cells (1 × 106/well) were seeded in 24-well plates in RPMI medium (with 10% FCS) followed by treatment with the indicated concentrations of doxorubicin for 16 h. Cell viability was then measured using Trypan blue reagent. Click here for file

Additional file 5: Table S1 Primers used for the cloning of deletion constructs. Click here for file

Additional file 6: Table S2 Primers used for the cloning of small deletion constructs. Click here for file

Additional file 7: Table S3 Primers used for the cloning of mutational analyses constructs. Click here for file
