# Supplementary figures and images for: Cold shock Y-box protein-1 proteolysis autoregulates its transcriptional activities
Source: Cell Commun Signal. 2013 Aug 27;11:63. doi: 10.1186/1478-811X-11-63 (PMC3766096; doi:10.1186/1478-811X-11-63)

Supplementary Figure 1

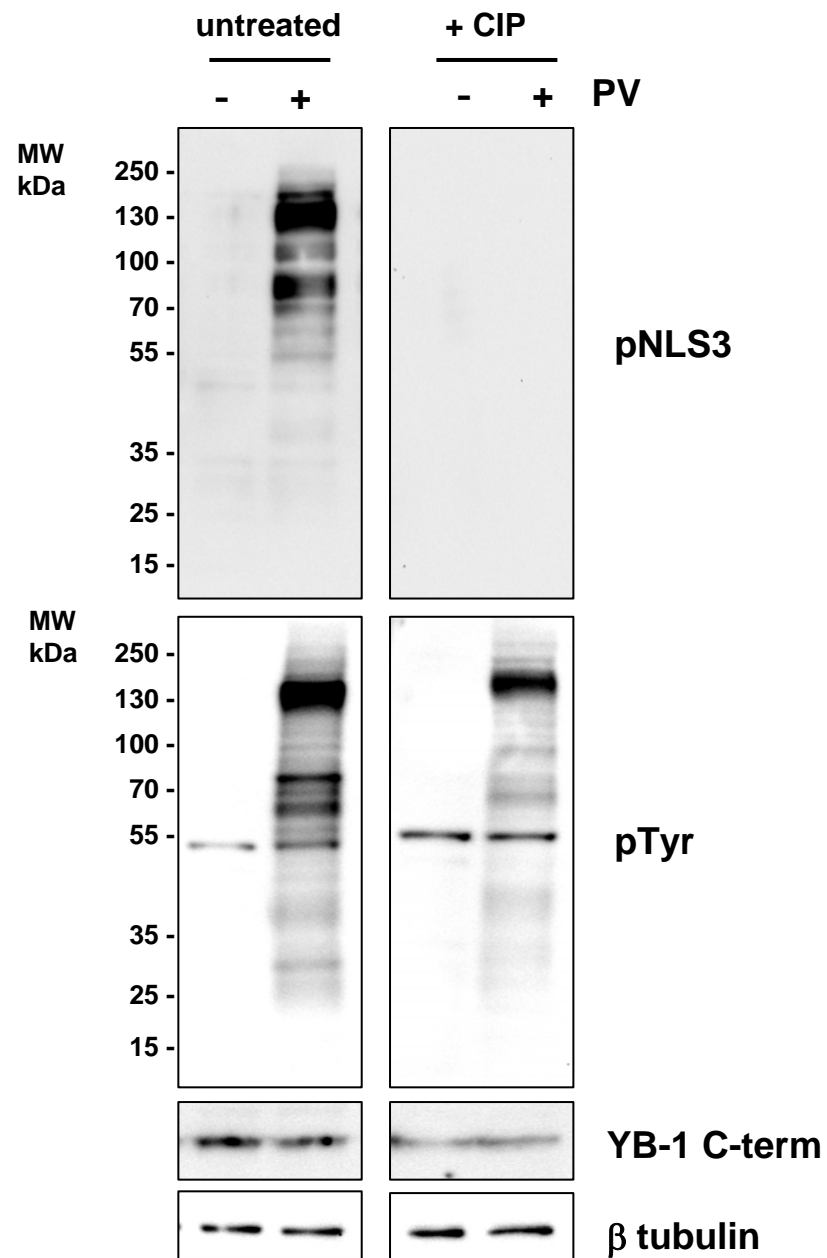

Supplement: Additional file 1: Figure S1 — Antibody specificity testing with and without calf intestinal alkaline phosphatase treatment. Rat mesangial cells were left untreated (-) or treated (+) with the protein tyrosine phosphatase inhibitor pervanadate (PV) to maximize phosphorylation. Cell lysates were loaded in duplicate, separated by SDS-PAGE, and transferred onto membranes. The membrane was cut and blocked. In addition, one membrane was incubated in alkaline phosphate buffer with 1U calf intestinal alkaline phosphatase (CIP)/ μg protein for 60 minutes (+CIP). The membranes were the incubated with primary antibody as indicated and visualized using ECL detection. As seen, CIP treatment completely ablates the signal detected using the pNLS3 antibody demonstrating its phospho-specificity. The pan-phosphotyrosine antibody [4G10] shows that not all tyrosine phosphorylation has been removed. The YB-1 and β-tubulin signals are comparable. The position of the protein standards and the relative molecular weight (MW) in kiloDaltons (kDa) are indicated. [file 1478-811X-11-63-S1.pdf]

**Supplementary Figure 2**

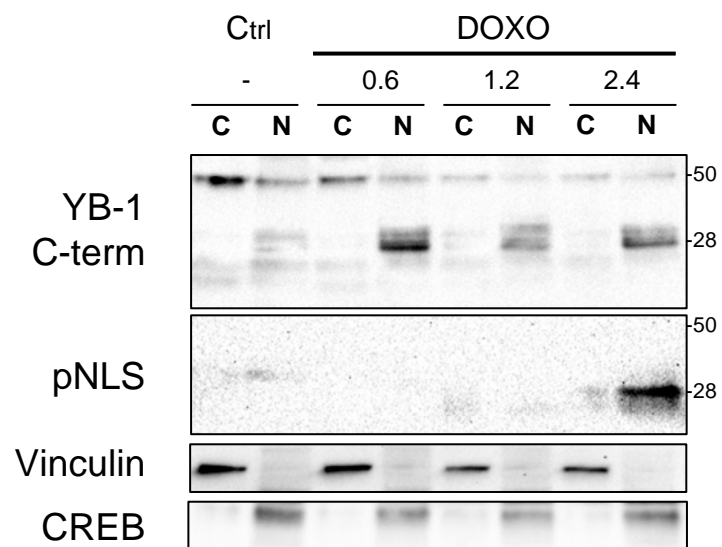

Supplement: Additional file 2: Figure S2 — Subcellular localization of YB-1 protein fragments following genotoxic stress. Immunoblotting of fractionated cell lysates from rat mesangial cells exposed to doxorubicin for 14 h at increasing concentrations (0.6 , 1.2, and 2.4 μg/ml). Cytoplasmic and nuclear proteins were separated and purity ascertained by detection of vinculin and CREB. Additionally, blotting with the pNLS3 antibody shows that the phosphorylated C-terminal fragment (p28) is found exclusively in the nuclear fraction. [file 1478-811X-11-63-S2.pdf]

Supplementary Figure 3

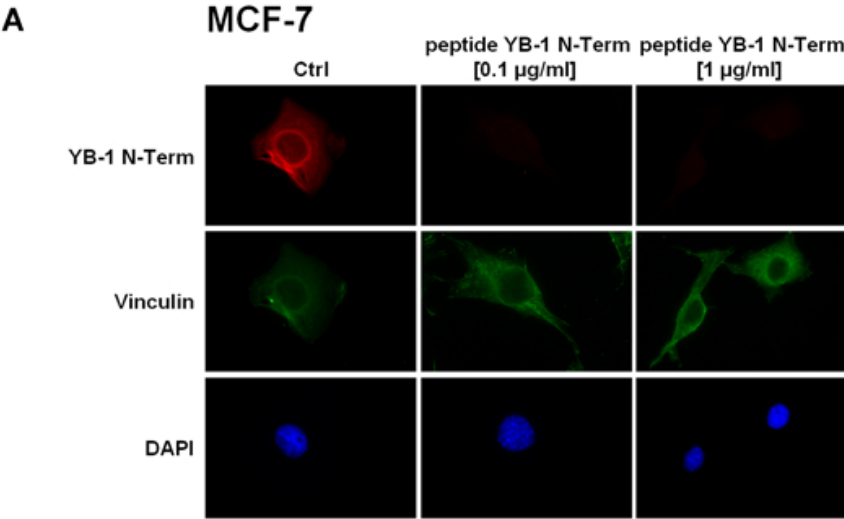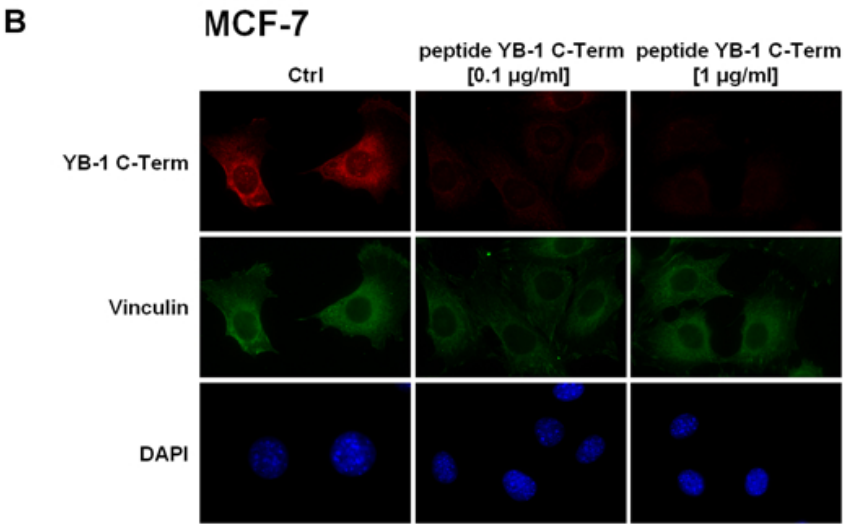

Supplement: Additional file 3: Figure S3 — Antibody specificity testing with preincubation of immunization peptides in MCF-7 cells. Distribution of endogenous YB-1 protein was assessed by immunofluorescence microscopy in MCF-7 cells with a peptide-derived affinity purified polyclonal YB-1 antiserum directed against the N-terminus (primary antibody). Upper left panel: untreated N-terminal antibody. Upper middle panel: antibody mixed with 0.1 μg/ml of immunizing peptide (YB-1 amino acids 21 to 37: SAADTKPGTTGSGAGSG). Upper right panel: antibody mixed with with 1 μg/ml of immunizing peptide. Middle panels: Murine anti-vinculin antibody was utilized to visualize the cell structure. Lower panels: Nuclei were visualized with DAPI. Images were taken at ×63 magnification. [file 1478-811X-11-63-S3.pdf]

Supplementary Figure 4

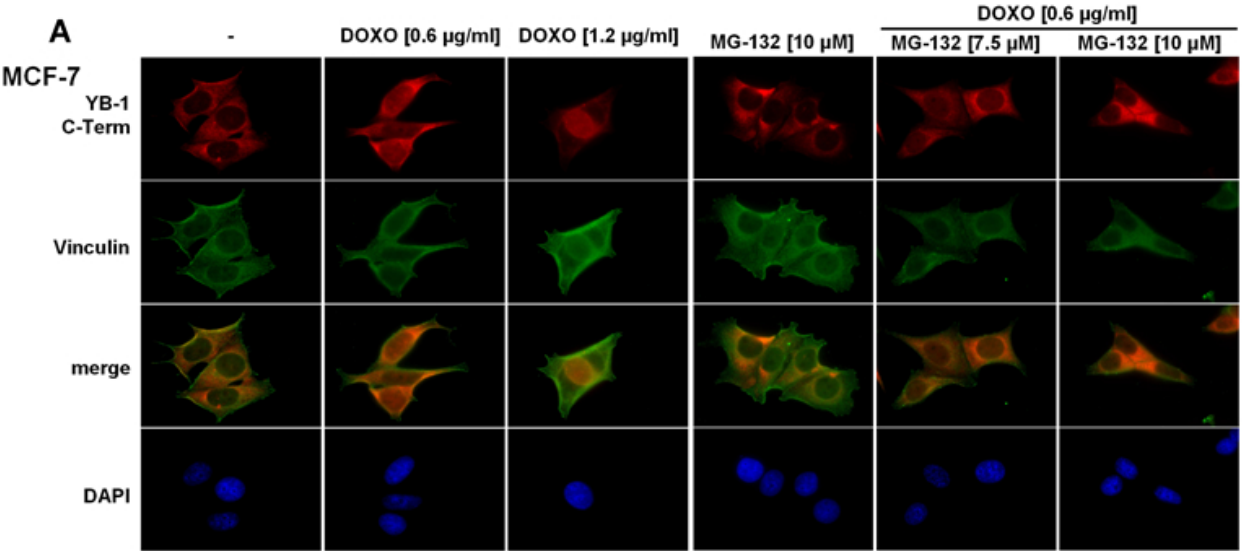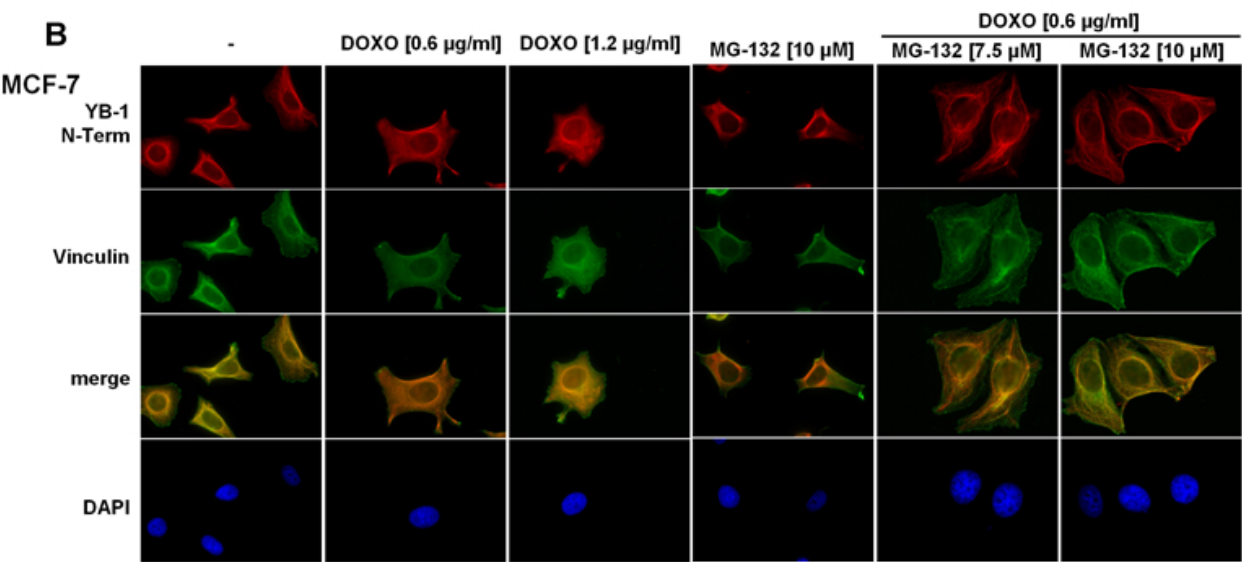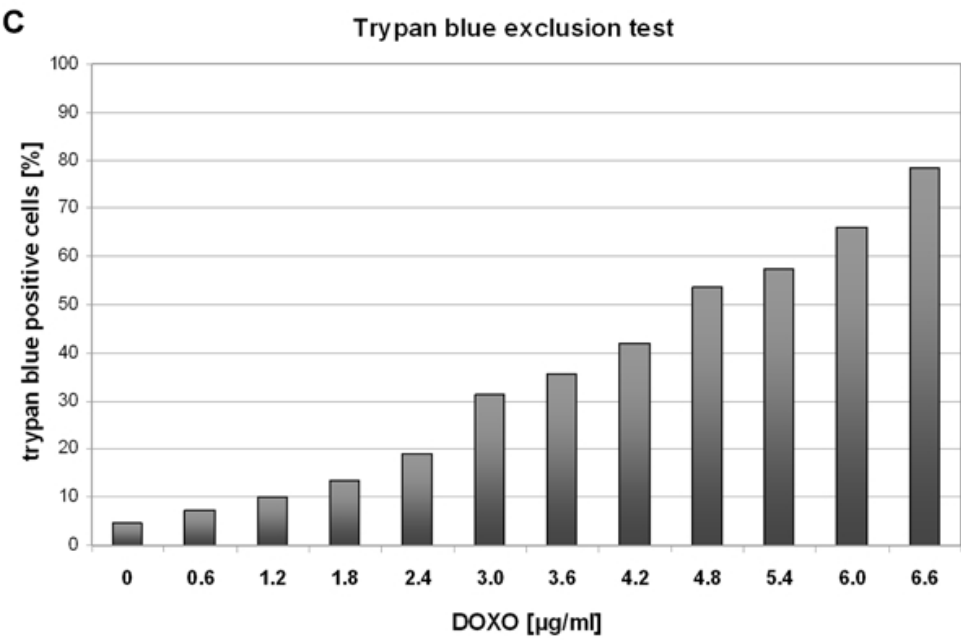

Supplement: Additional file 4: Figure S4 — Subcellular localization of YB-1 protein fragments following genotoxic stress in the absence and presence of proteasomal inhibitor in MCF-7 breast cancer cells. 1 2. A. Distribution of endogenous YB-1 protein was assessed by immunofluorescence microscopy in MCF-7 cells following immunodetection with the anti-YB-1 antiserum directed against the C-terminus (primary antibody). Murine anti-vinculin antibody was utilized to visualize cell structures. Fluorescence labelled secondary antibodies consisted of anti-rabbit IgG(Fab)-Cy3 and anti-mouse IgG(Fab)-FITC. Nuclei were visualized by DAPI staining. MCF-7 cells were incubated for 16 h with doxorubicin at increasing concentrations (0.6 and 1.2 μg/ml) in the absence or presence of proteasome inhibitor MG-132 (7.5 and 10 μmol/l). Images were taken at ×63 magnification. B. Distribution of endogenous YB-1 protein was assessed by immunofluorescence microscopy in MCF-7 cells according to the protocol outlined in A with polyclonal YB-1 antiserum directed against the N-terminus (N-Term, primary antibody). C. Cytotoxicity assay with increasing concentrations of doxorubicin. Rat mesangial cells (1 × 10^6^/well) were seeded in 24-well plates in RPMI medium (with 10% FCS) followed by treatment with the indicated concentrations of doxorubicin for 16 h. Cell viability was then measured using Trypan blue reagent. [file 1478-811X-11-63-S4.pdf]
