# Supplementary material for: Cold shock Y-box protein-1 proteolysis autoregulates its transcriptional activities
Source: Cell Commun Signal. 2013 Aug 27;11:63. doi: 10.1186/1478-811X-11-63 (PMC3766096; doi:10.1186/1478-811X-11-63)
Supplement: Additional file 5: Table S1 — Primers used for the cloning of deletion constructs. [file 1478-811X-11-63-S5.pdf]

Supplementary Table 1: Primers used for the cloning of deletion constructs

Construct	Sense Primer	Antisense Primer
P 1-57	CGGAATTCCGATGAGCAGCGAGGCCGAGA	CGGGATCCCGCTTCGTTGCGATGACCTTCTT
P 52-101	GGAATTCCAAGAAGGTCATCGCAACGAA	CGGGATCCCGTGCGAAGGTACTTCCTGGGG
P 96-146	CGGAATTCCGATGCCCAGGAAGTACCTTCGCAG	CGGGATCCCGTCTATAATGGTTACGGTCTGCTGC
P 52-146	GGAATTCCAAGAAGGTCATCGCAACGAA	CGGGATCCCGTCTATAATGGTTACGGTCTGCTGC
P 242-292	GAAGATCTTCGGGATATAGACCACGATTCCGC	GGAATTCCTGGGCGTCTGCGTCGGTAAT
P 69-146	GGAATTCCATGAGGAACGGATATGGTTTCATC	CGGGATCCCGTCTATAATGGTTACGGTCTGCTGC
P 89-146	GGAATTCCATGACTGCCATAAAGAAGAATAACC	CGGGATCCCGTCTATAATGGTTACGGTCTGCTGC
pCTF	GAAGATCTTCATGGAGGGTGCTGACAACCAG	GAAGATCTTCATGGAGGGTGCTGACAACCAG
pSG5 (CTF)	CGGGATCCCGATGGAGGGTGCTGACAACCAG	GAAGATCTTCTTACTCGGGAGCGGACGAATTCTC
