# Supplementary material for: Cold shock Y-box protein-1 proteolysis autoregulates its transcriptional activities
Source: Cell Commun Signal. 2013 Aug 27;11:63. doi: 10.1186/1478-811X-11-63 (PMC3766096; doi:10.1186/1478-811X-11-63)
Supplement: Additional file 6: Table S2 — Primers used for the cloning of small deletion constructs. [file 1478-811X-11-63-S6.pdf]

Supplementary Table 2: Primers used for the cloning of small deletion constructs

Construct	Sense Primer	Antisense Primer
P 149-156	GATCTCCACGTCGTAGGGGTCCTCCACGCG	AATTCGCGTGGAGGACCCCTACGACGTGGA
P 185-194	GATCTCGCCGGCCCTACCGCAGGCGAAGGTTCCCAG	AATTCTGGGAACCTTCGCCTGCGGTAGGGCCGGCGA
P 243-249	GATCTCCACGATTCCGCAGGGGCCCTG	AATTCAGGGCCCCTGCGGAATCGTGGA
P 276-292	GATCTCCACCTCAACGTCGGTACCGCCGCAACTTCAATTACCG ACGCAGACGCCCAG	AATTCTGGGCGTCTGCGTCGGTAATTGAAGTTGCGGCGGTACCGACGTTGAGGTGGA
P 10-30	AATTCATGCCGCCCGCCGCCCCCCCCGCCGCCCCCGCCCTCAGCGCCGCCGACACCAAGCCCGGCACTACG	GATCCGTAGTGCCGGGCTTGGTGTCGGCGGCGCTGAGGGCGGGGGCGGCGGGGGGGGCGGCGGGCGGCATG
P 10-20	AATTCATGCAGCGAGGCCGAGACCCAGCAGCCGCCCGCCGCCCCCCCCGCCGCCCCCGCCCTCAGG	GATCCCTGAGGGCGGGGGCGGCGGGGGGGGCGGCGGGCGGCTGCTGGGTCTCGGCCTCGCTGCTCATG
P 30-50	AATTCATGGGCAGCGGCGCAGGGAGCGGTGGCCCGGGCGGCCTCACATCGGCGGCGCCTGCCGGCGGGGACAAGAAGGTCATCGCAACGAAGGTTTTG	GATCCAAAACCTTCGTTGCGATGACCTTCTTGTCCCCGCCGGCAGGCGCCGCCGATGTGAGGCCGCCCGGGCCACCGCTCCCTGCGCCGCTGCCCAGG
P 21-40	AATTCATGGCGCCGCCGACACCAAGCCCGGCACTACGGGCAGCGGCGCAGGGAGCGGTGGCCCGGGCGGG	GATCCCCGCCCGGGCCACCGCTCCCTGCGCTGCCCGTAGTGCCGGGCTTGGTGTCGGCGGCGCTCATG
P 41-60	AATTCATGGGCCTCACATCGGCGGCGCCTGCCGGCGGGGACAAGAAGGTCATCGCAACGAAGGTTTTG	GATCCAAACCTTCGTTGCGATGACCTTCTTGTCCCCGCCGGCAGGCGCCGCCGATGTGAGGCCCATG
P 89-109	AATTCATGACTGCCATAAAGAAGAATAACCCCAGGAAGTACCTTCGCAGTGTAGGAGATGGAGAGACTGTG	GATCCACAGTCTCTCCATCTCCTACACTGCGAAGGTACTTCCTGGGGTTATTCTTCTTTATGGCAGTCATG
P 100-109	ATTTCATGCTTCGCAGTGTAGGAGATGGAGAGACTGTG	GATCCACAGTCTCTCCATCTCCATCACTGCGAAGCATG
