# Supplementary material for: Cold shock Y-box protein-1 proteolysis autoregulates its transcriptional activities
Source: Cell Commun Signal. 2013 Aug 27;11:63. doi: 10.1186/1478-811X-11-63 (PMC3766096; doi:10.1186/1478-811X-11-63)
Supplement: Additional file 7: Table S3 — Primers used for the cloning of mutational analyses constructs. [file 1478-811X-11-63-S7.pdf]

Supplementary Table 3: Primers used for the cloning of mutational analyses constructs

Construct	Sense Primer	Antisense Primer
P_{NLS}185 I	GATCTCGCAGGCGAAGGG	AATTCCCTTCGCCTGCGA
P_{NLS}185 II	GATCTCCCTACCGCAGGCGAAGGTTCG	AATTCGAACCTTCGCCTGCGGTAGGGA
P_{NLS}185 III	GATCTCGCGGGCCCTACCGCAGGCGAAGGTTCCCAG	AATTCTGGGAACCTTCGCCTGCGGTAGGGCCCGCGA
P_{NLS}185 IV	GATCTCGCCGGCCCTACCGCGGGCGAAGGTTCCCAG	AATTCTGGGAACCTTCGCCCGCGGTAGGGCCGGCGA
P_{NLS}185 T/P	GATCTCGCCGGCCCTTCCGCAGGCGAAGGTTCCCAG	AATTCTGGGAACCTTCGCCTGCGGAAGGGCCGGCGA
P_{NLS}276 I	AATTCATGCCACCTCAACGTCGGTACCGCCGCCCG	GATCCGGGCGGCGGTACCGACGTTGAGGTGGCATG
P_{NLS}276 II	AATTCATGCGACGCAGACGCCCACCG	GATCCGGTGGGCGTCTGCGTCGCATG
P_{NLS}276 III	AATTCATGCCACCTCAACGTCGGTACCGCCGCCGACGCAG ACGCCCACCG	GATCCGGTGGGCGTCTGCGTCGGCGGCGGTACCGACGTTG AGGTGGCATG
P_{NLS}276 IV	AATTCATGCCACCTCAACGTCGGTACCGCCGCGGCAACTT CAATTACGGCCGACGCAGACGCCCACCG	GATCCGGTGGGCGTCTGCGTCGGCCGTAATTGAAGTTGCC GCGGCGGTACCGACGTTGAGGTGGCATG
P_{NLS}276 V	AATTCATGCCACCTCAACGTCGGTTCCGCCGCAACTTCAAT TACCGACGCAGACGCCCACCG	GATCCGGTGGGCGTCTGCGTCGGTAATTGAAGTTGCGGCG GAACCGACGTTGAGGTGGCATG
P_{NLS}276 VI	AATTCATGCCACCTCAACGTCGGTACCGCCGCAACTTCAAT TTCCGACGCAGACGCCCACCG	GATCCGGTGGGCGTCTGCGTCGGAAATTGAAGTTGCGGCG GTACCGACGTTGAGGTGGCATG
